# Graphene in Water is Hardly Ever Neutral

**DOI:** 10.1002/advs.202403760

**Published:** 2024-08-19

**Authors:** Luna Boulbet‐Friedelmeyer, Gilles Pécastaings, Christine Labrugère‐Sarroste, Jordi Faraudo, Alain Pénicaud, Carlos Drummond

**Affiliations:** ^1^ Univ. Bordeaux CNRS CRPP UMR 5031 Pessac 33600 France; ^2^ Carbon Waters 14 avenue Pey Berland Pessac 33600 France; ^3^ Université de Bordeaux CNRS PLACAMAT UAR 3626 Pessac F‐33600 France; ^4^ Institut de Ciència de Materials de Barcelona (ICMAB‐CSIC) Campus de la UAB Bellaterra E‐08173 Spain

**Keywords:** 2D‐materials, DFT calculations, graphene dispersions, raphene

## Abstract

Graphene in water is electrically charged in most conditions. The level of charge can be large enough to stabilize single (or few) layer graphene colloidal dispersions in water, without the need of using any other additive. In this work, potentiometric titration, isothermal titration calorimetry, electrokinetic measurements, Density Functional Theory calculations, Raman Spectroscopy, and direct force measurements using Atomic Force Microscopy to investigate this charge and explore its origin are combined. The body of data collected suggests that this charge is a consequence of the interaction between water ions (hydroxide and hydronium) and graphene, and can be conveniently tuned (in magnitude and sign) by changing the pH of water.

## Graphical abstract

Stable dispersion of micrometer‐sized graphene flakes in water can be produced by solvent substitution from graphite intercalation compound solutions. The favorable interaction between graphene and hydroxide ions from water self‐ionization prompts the electrostatic repulsion between graphene flakes in water. The stability of the graphene aqueous dispersions results from the interplay between this repulsive electrostatic interaction and the attractive van der Waals forces.

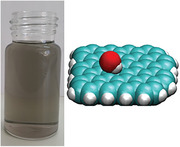

## 1 Introduction

The prospects of graphene as a “wonder material” have been extensively discussed in the literature. In this sense, a large number of potential applications of graphene aim for its use as an additive in composites or as a part of complex formulations. Nevertheless, before it can achieve its full potential, reliable, large‐scale methods of production must be developed. For these purposes, it is convenient to produce stable graphene dispersions/solutions. However, graphene is hardly soluble in any solvent, and its liquid dispersion often requires the use of stabilizers and extensive shearing, with potentially negative consequences (e.g., introduction of defects). As a possible path to overcome these limitations, we have recently shown that spontaneous (albeit slow) dissolution of graphite intercalation compounds GIC, followed by solvent transfer, can be a sensible strategy to produce surfactant‐free graphene dispersion in water.^[^ ^1^ ^]^ In virtue of the presence of the intercalants in GICs, extensive exfoliation is readily achieved upon dissolution.^[^ ^2^, ^3^, ^4^, ^5^ ^]^ We have shown that after solvent exchange has been completed, single‐layer graphene remains stable in water without the need for adding any stabilizing agent.^[^ ^1^ ^]^ We, and others, have shown that repulsive electrostatic forces, together with the less significant destabilizing dispersion forces due to its high‐aspect‐ratio (length/thickness), are the reasons for the stability of aqueous dispersions of graphene^[^ ^1^, ^6^, ^7^, ^8^ ^]^ and reduced graphene oxide rGO.^[^ ^9^, ^10^ ^]^ While the electrostatic stabilization of the additive‐free graphene aqueous dispersion seems verified, the physical reasons behind the charge at the graphene‐water interface are still not conclusively established.

It is often described with surprise that hydrophobic objects in water carry a pH‐dependent net charge, which is of negative sign in basic environments. Reports on this phenomenon abound in the literature after early electrophoretic studies of bubbles and hydrocarbon drops were described.^[^ ^11^, ^12^, ^13^ ^]^ The source of this charge has fueled a large number of debates in the literature.^[^ ^14^, ^15^ ^]^ Nevertheless, the great majority of interfaces are in fact electrically charged, as the very existence of an interface often produces a (local) break of electroneutrality. In contact with an electrolyte, a potential difference is generally observed between a metallic electrode and the surrounding solution (often called open circuit potential). In fact, the condition of no charge at the interface is rather exceptional, and it is identified as a prominent point: the potential of zero charge, PZC, which designates the potential at which the net electrode charge density vanishes.^[^ ^16^ ^]^ Imposing on the electrode a potential other than the PZC (i.e., by using a potentiostat) forces the occurrence of a non‐null charge density at the interfacial region. Somehow analogously, for many interfaces, the pH of zero charge (pH_0_) is defined as the pH at which its (average) interfacial charge density vanishes. Above (below) this pH, the surface gets negatively (positively) charged. pH_0_ is often perceived as a surface property, but in reality, it is more appropriate to describe it in terms of the global interface, as it depends not only on the type of surface but also on the properties of the solution (concentration and type of electrolyte). As the value of pH_0_ determines many properties of the materials (e.g., ionic adsorption, dispersion stability, homo‐ or heterocoagulation) precise protocols for its determination have been established.^[^ ^16^ ^]^

As mentioned above, it has been extensively documented that many hydrophobic‐water interfaces show a pH‐dependent interfacial charge, with pH_0_ between 3 and 5. However, there is still no consensus on the origin of the observed electrical charge. Similar uncertainty arises in the case of graphene‐water interfaces: as mentioned above, the reasons behind their charge are not established with certainty, although several hypotheses have been advanced. It has been suggested that the adsorption of water ions (in particular OH^−^) could explain the negative zeta potential generally observed in graphene dispersed in water.^[^ ^1^, ^8^ ^]^ This hypothesis has often been evoked by the community studying hydrophobic‐water interfaces. Other groups have proposed that the presence of ionizable functional groups (e.g., carboxylic acids) is responsible for the negative charges often observed. This hypothesis is particularly appealing in the case of rGO.^[^ ^7^, ^9^, ^10^ ^]^ Both scenarios explain the pH dependence of the charge and stability of graphene dispersions. It has also been proposed that direct charge transfer from water (or water ions) could be at the origin of the often observed negative charge at hydrophobic‐water interfaces.^[^ ^17^, ^18^ ^]^ Finally, it has recently been suggested that the presence of bicarbonate anions, which originated from CO_2_ dissolution, could also be responsible for the negative charges at hydrophobic interfaces.^[^ ^19^ ^]^ Understanding the origin of the charge of graphene in water should drive the design of strategies for tuning the stability of aqueous graphene dispersions. In this work, we have investigated the electrostatic potential of the graphene‐water interface, combining experimental strategies at different length scales, from bulk to nanometric level, with Density Functional Theory DFT calculations, in an attempt to elucidate the fundamental reasons for the observed charge. All our results are consistent with a pH‐dependent charge of graphene in water, which appears to be a consequence of the favorable interaction between water ions (hydroxide and, to a lesser extent, hydronium) and graphene.

## 2 Results and Discussion

### 2.1 System Description

EdG samples were prepared as described in the previous section. Exposure of KC_8_ to THF leads to the dissolution of graphenide (C_8_^−^)_∞_ and associated counterions K^+^. Upon exposure to air, graphenide is oxidized (loss of electrons) to neutral graphene whereas oxygen is reduced to superoxide O_2_^−^.^[^ ^20^ ^]^ Further exposure to water and /or humidity leads to KOH.^1^ After preparation, a dark‐colored water suspension was obtained. In agreement with our earlier studies, they remained stable for months stored at 5 °C, without showing signs of aggregation or destabilization. Typical pH values of EdG samples varied between 8.4 and 9. Interestingly, similar pH values were measured after the removal of the graphene from the liquid phase by filtration using a 0.02 µm PTFE filter.

To assess the degree of functionalization of graphene during the production of EdG, we have explored deposits prepared as described above, by XPS. The most significant information is the variation of the amount of oxygen in the sample compared with the one in the graphite used for the synthesis, due to the addition of different functional groups during the oxidation of the graphenide sample and transfer to water. As can be observed in Figure S1 (Supporting Information), after the outmost layer is slowly sputtered away, the oxygen content in the sample is marginally larger than the amount observed in the graphite used to prepare the samples. We can not determine if this evolution comes only from spurious contamination of the outer layer from exposure to the environment, or if it also evidences the surface enrichment in oxygenated material due to the coating process. After sputtering, graphene showed only a small shoulder on the high‐energy side of the C1s peak, indicating some sp^3^ carbon functionalization (Figure S2, Supporting Information).

As an additional gauge of the quality of the prepared samples, EdG Raman spectra measured directly (vide infra) showed all the features expected for graphene of good quality (narrow 2D bands, large I_2D_/I_G_ ratios, and a D band corresponding to an estimated amount of sp^3^ defects less than 1/1000), as we have discussed extensively in the past.^[^ ^21^ ^]^

### 2.2 Macroscopic Scale: Acid‐Base Titration of EdG

**Figure 1:**
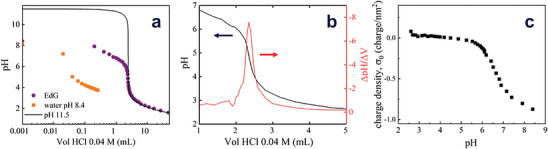
a) Potentiometric titration of EdG (violet circles) and water at pH 8.4 (orange squares). The pH is reduced by successive additions of aliquots of 0.04 M HCl. The continuous line represents the expected response for the titration of water at pH 11.4. b) Titration data for EdG (same data as a) in linear representation) and the first derivative of this data, to evaluate the equivalence point. c) pH dependence of the apparent charge density of graphene in EdG, evaluated as described in the text.

We measured the variation of the pH of EdG (measured directly on EdG) after the addition of aliquots of concentrated HCl, as described in the experimental section. For the EdG sample investigated, the measured pH of freshly prepared EdG was 8.4. A typical titration curve obtained by this procedure is reported in **Figure** **1**a. Several aspects are noteworthy. First, the initial pH variation with acid addition is much smaller than what is observed in a blank titration (a sample of DI water with a pH value initially adjusted to 8.4, by adding a minute amount of base). EdG titration data bear a resemblance to what could be expected upon titration of a weak base with a strong acid: initially, the pH drops slowly as acid is added; then, as an equivalence point is approached, we observed a large drop in pH with a small acid addition. As expected, as we got past the equivalence point, the pH continued dropping with further addition of acid.

Given that we are studying a rather heterogeneous and ill‐defined material, it is significant that a sharp transition with a clearly defined equivalence point can be distinguished, at a pH ca. 4.86 (2.35 mL of added HCl). From the first derivative data shown in Figure 1b, it can be gauged that a single equivalence point is present on the titration curve. This is in strong contrast with the great majority of titration curves reported for graphene aqueous dispersions (mostly obtained with reduced graphene oxide, rGO), which shows broad titration signals with multiple inflection points.^[^ ^10^, ^22^, ^23^ ^]^ Furthermore, no hint of the conversion of carbonate into bicarbonate, which would appear as a rapid pH change ca. 8.5, is detected. We can attempt to rationalize the observed data in terms of the titration of a weak base, *A^−^*, (the conjugate base of a weak acid, *AH*) with a strong acid, as:

(1) $$A^{-}+H_{3}O^{+}\;↔\;AH+H_{2}O\;\;\;\;\;\;\;K_{a}=\frac{\left[H_{3}O^{+}\right]\left[A^{-}\right]}{\left[AH\right]}\;$$

In the titration curve, the *pK_a_* of the acid would be similar to the pH at the half‐equivalence point, *pH_1/2eq_*, when the concentrations of the weak acid, *AH*, and the conjugate base, *A^−^*, are similar (*pH_1/2eq_ = pK_a_*). Thus, our data indicate a *pK_a_* value close to 6.7. This well‐defined value appears to be either too large, compared with values corresponding to carboxylic acids (typically between 3.5 and 5), or too small, compared with phenols (typically between 9 and 10), the typical functional groups that could result from graphene oxidation. The sharpness of the transition and the uncommon *pK_a_* observed, suggest that a different interpretation of the data is required. It is noteworthy that *pK_a_* is remarkably close to neutral pH, the equivalence point of the titration of a strong acid with a strong base.

The most striking feature of the titration curve is the large amount of acid required to neutralize the sample of EdG, given the low initial pH value. Several studies of surfactant‐free emulsions have postulated that significant emulsion stability can be achieved by the adsorption of OH^−^ ions at the oil‐water interface. These studies have pointed out a significant inconsistency between the pH measured by conventional potentiometry and the amount of hydroxide effectively present in the samples. This difference was explained by a change of the effective pH in the continuous phase, due to hydroxide adsorption at the oil‐water interface.^[^ ^24^, ^25^, ^26^ ^]^ In the same line of thought, we can attempt an alternative interpretation of our data in terms of a strong base‐strong acid titration, consistent with the distinctive equivalence point observed. We can readily calculate the expected titration curve, from the observed equivalence point (which should occur at pH 7.0), displayed as a continuous line in Figure 1. As can be observed in the figure, this representation describes well the data measured in the acidic part of the titration curve but diverges on the basic side. In this scenario, the pH of the pristine EdG sample should be 11.4, instead of the measured value of 8.4. An independent estimation of the [OH^−^] in the sample of EdG studied can be obtained from the preparation conditions. The concentration of graphene in this sample was 0.5 g L^−1^. As described in the experimental section, EdG samples are prepared from the oxidation of graphene solutions (spontaneous dissolution of the KC_8_ salt in THF). After complete graphene oxidation and transfer to water, the main byproduct of the reaction is hydroxide anions OH^−^, which remain in the aqueous environment (with K^+^ as counterion). Assuming a perfect conversion of KC_8_ to KOH and neutral C (graphene), a concentration of OH^−^ in the final dispersion of 5.2 mM can be anticipated. Assuming a value of 1 to the activity coefficient of the proton, this would correspond to a pH of 11.7, remarkably close to the value of 11.4 extrapolated from the titration curve. The coherence between the 2 pH values is noteworthy, given the level of uncertainty in the different experimental steps. This result strongly suggests that hydroxide ions at sufficiently large concentrations are indeed interacting with graphene in the aqueous dispersion, affecting the potential of the glass membrane of the electrode. We can then use the potentiometric titration data to obtain a crude estimation of the charge density on graphene, *σ_0_*, in EdG. For the titration of the basic EdG suspension with a strong acid, it can be shown that^[^ ^27^ ^]^

(2) $$\sigma_{0}\;=F\cdot \left(\Gamma_{H^{+}}-\Gamma_{OH^{-}}\right)\;=\frac{F\cdot c_{\mathrm{HCl}}\left(\upsilon_{b}-\upsilon_{\mathrm{HCl}}\right)}{s\cdot m\cdot V}\;$$

where *Γ*_H_ *_+_* and *Γ*_OH_ *_‐_* are the specific surface concentration of protons and hydroxide at a given pH condition, *c*_HCl_ is the concentration of the titrant solution (HCl), *s* is the specific surface area of graphene, *m* the concentration of graphene in the solution, *V* the volume titrated, *υ_b_* and *υ*_HCL_ and are the volume of titrant added for obtain a given pH value in absence (blank titration) and presence of graphene particles, and *F* the Faraday constant.

By using Equation (2), we can evaluate the charge density changes from the titration data, as presented in Figure 1c. There are 2 main complications with this analysis. First, as we measured the difference between the blank and the EdG titrations, we could only calculate the variation in charge density (instead of the absolute value). Nevertheless, we can make the hypothesis that the actual neutrality condition is close to the flat segment observed in Figure 1c, at intermediate pH values. As *σ_0_* varies very gradually in this range of pH, this appears as a reasonable assumption. More problematic is the determination of the specific surface area, *s*, which depends on the particular exfoliation conditions of graphene in EdG. For the sake of the analysis, we can assume full graphene exfoliation in EdG. Despite the uncertainty introduced by these approximations, we can draw some interesting conclusions from this data representation. First, it seems apparent that the neutrality condition of graphene in EdG is far below neutral pH: the point of zero charge appears to be between pH 4 and 5, which indicates a preference for graphene for hydroxide ions than for protons. Second, the apparent *σ_0_* appears to change more significantly under basic than under acidic conditions. These 2 conclusions are independent of the hypothesis formulated. This result depends on the assumed specific area of graphene, determined by the exfoliation of the material. Under the assumption of full exfoliation, a large value of *σ_0_* emerges (ca. −0.9 charge nm^−2^ corresponding to a mean separation of 0.6 nm between charges). This value is probably overestimated: by using Equation (2), we are implicitly assuming that the measured pH is equal to ‐log[H^+^] in the continuous aqueous phase (i.e., the activity coefficient of the hydronium ions is equal to 1), which may lead to the use of a wrongly low OH^−^ concentration in the blank titration.

We can summarize here the different pieces of information gathered from the potentiometric titration just described. The low initial pH of EdG (or its continuous phase upon filtration), and the large amount of concentrated acid required to neutralize the system, allow only 2 possible explanations. Either there is a large amount of acidic functional groups on the material, or there is substantial interaction between the graphene and hydroxide ions. The first hypothesis appears unlikely, given the sharpness of the transition, the estimated values of *pK_a_*, and the small amount of oxygen content measured by XPS. As will be discussed in the following sections, this hypothesis is also incompatible with the results of ITC and Raman.

### 2.3 Macroscopic Scale: ITC Study of EdG

**Figure 2:**
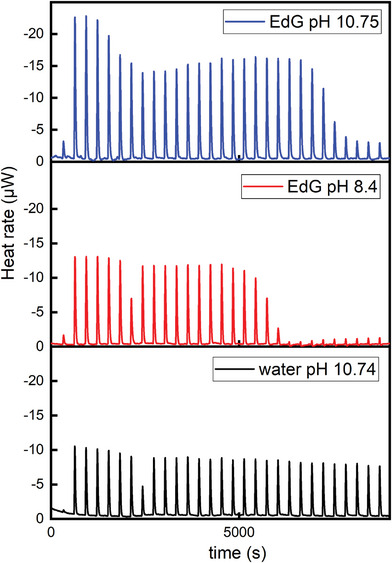
ITC enthalpograms of water at pH 4.06 titrated with basic water and EdG, as indicated. T = 25C (The curves have been vertically shifted for clarity).

As described in the experimental section, we performed a number of ITC experiments to investigate the heat exchanged upon adding aliquots of EdG (0.1 g L^−1^) to different aqueous systems. Typical ITC curves are presented in **Figure** **2**. They correspond to the titration of acid water (titrand, pH 4.06) by adding basic water or EdG (titrant). All the curves were measured following the same experimental protocol, as described above.

Exothermic heat exchange was observed in the titration of water at basic pH (pH 10.75) on water at acid pH (pH 4.06), as could be anticipated. The measured enthalpy change observed (55.7 kJ moL^−1^ of OH^−^ neutralized) is coherent with the neutralization heat of a strong base (KOH) reacting with a strong acid (HCl).^[^ ^28^, ^29^ ^]^ As can be observed in the Figure, the enthalpy change of each injection is mostly unchanged during the run, signaling that the equivalence point was not reached at the end of the experiment (after 30 injections of basic water). The results are substantially different upon injection of EdG (pH 8.4) into the same acidic solution. Several effects can be noted. First, the enthalpy change for each injection is substantially enhanced at the beginning of the titration. It is noticeable that the heat released at each injection of EdG (pH 8.4) is 1.5 times larger than the one observed with water at pH 10.75 (362 vs 239 µJ injection^−1^). Second, an equivalence point appears to be reached after a number of injections (≈20 injections in the data set reported). The pH value at this equivalence point was 4.25. Finally, the heat exchange after the equivalence point has been reached is substantially reduced, and is similar to the values observed when mixing neutral water (background titration). It is noteworthy that negligible enthalpy changes are observed upon titration of water at pH 8.4 with the same acid solution. In the third titration reported in the Figure, the pH of EdG has been increased by base addition, to reach a value similar to the one measured for the case of pure basic water (pH 10.75). In this case, several changes can be noted. As for pure EdG, an equivalence point can be identified. However, the heat exchange at each injection before the equivalence point is substantially larger: ca. 544 µJ injection^−1^ for EdG at pH 10.75, vs 239 µJ injection^−1^ for water at similar pH. Interestingly, the difference in enthalpy change between the 2 EdG samples (basic vs plain EdG) is close to the heat of neutralization of the base added to basify the EdG. In both cases of EdG titration, the pH of the mixture at the equivalent point is ca. 4.25, as determined by performing identical titrations outside of the ITC setup. Important implications can be inferred from the ITC data described. The enthalpy changes observed by ITC appear incompatible with the neutralization of weak acidic groups (e.g., carboxylic acids or phenol groups). If we consider the process as a strong base‐strong acid neutralization, the pH of EdG (defining pH as –log[H^+^]) would be 10.9, substantially larger than the measured value of 8.4. Interestingly, if we assume again perfect conversion of KC_8_ to KOH and neutral graphene, a pH 11.0 for the EdG 0.1 g L^−1^ can be estimated (if *f *= 1). It is also remarkable that the neutralization point, ca. pH 4.25, is far from neutral pH, in agreement with the results of the potentiometric titration.

### 2.4 Macroscopic Scale: Streaming Potential of EdG Deposits

**Figure 3:**
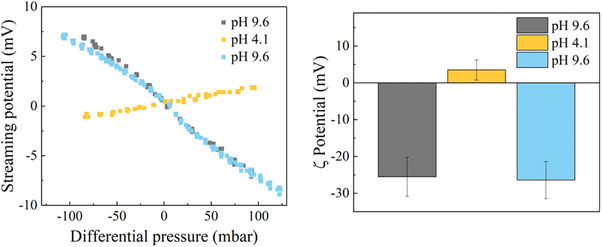
a) Streaming potential *ΔE_s_* as a function of applied pressure difference and b) *ζ* calculated from *ΔE_s_* data, for graphene from EdG deposited on rectangular pieces cut from a silicon wafer. Grey and blue data points/bars correspond to measurement at basic conditions before and after exposure to an acidic environment, to illustrate the reversibility of the measured potential.

The pH changes associated with the different titrations described in the previous section prompted the destabilization of graphene in EdG. For this reason, it was not possible to evaluate the reversibility of the pH‐induced changes in the material by those methods. To verify this aspect, other techniques to explore the electrostatic state of graphene in EdG were implemented. As described in the experimental section, we evaluate the potential zeta of graphene deposits by measuring the streaming potential. In these experiments, graphene deposits are exposed to a large amount of KCl solutions at controlled pH. If we discard the presence of charged functional groups in the graphene, the detected charges on the material will necessarily be due to the ionic adsorption (from the aqueous environment). When an applied pressure gradient *ΔP* is used to force the flow of an electrolyte solution through a channel with charged walls, a potential difference (streaming potential, *ΔE_s_*) is generated by the accumulation of the excess charges near the wall that are carried along by the liquid.^[^ ^16^ ^]^ As described in the experimental section, we measured *ΔE_s_* at different pressure drop values and different pH conditions and calculated the zeta potential of the graphene deposit, *ζ*, by using the Smoluchowski equation,^[^ ^16^ ^]^

(3) $$\zeta \;=\frac{\Delta E_{S}.\eta .k_{L}}{\varepsilon_{w}.\varepsilon_{0}.\Delta p}\;$$

where *ε_0_* is the electric permittivity of the vacuum, and *ε_w_*, *η*, and *k_L_* are the relative permittivity, viscosity, and conductivity of the solution. Typical results of *ΔE_s_* and *ζ*, are presented in **Figure** **3**. At low applied pressures, we generally observed a linear relationship between the applied pressure and the measured potential drop, indicating that we can apply Equation (3) to analyze our data. In agreement with the titration data, we observed a very small (positive) value of *ζ *acid pH and a significant negative value under basic conditions. More importantly, we found good reversibility and reproducibility in the measured *ζ *values upon repeatedly cycling pH. This result suggests that the graphene surface potential measured in these tests is an equilibrium value, and strongly contradicts the possibility of the negative charge on graphene being a consequence of spurious contamination or incomplete electron removal. In those scenarios, reversibility of the measured *ζ *upon pH cycling would not be likely.

### 2.5 Mesoscopic Scale: Single Flake Electrophoresis

The streaming potential tests just described were measured in surface deposits produced from EdG, which may introduce modifications to the material, and may be influenced by the supporting surface (silicon wafers). For these reasons, it may be of interest to evaluate the potential of graphene flakes in aqueous environment. The electrophoretic mobility *µ* of graphene flakes was measured for various pH values (electrokinetic titration) in media of different KCl concentrations. A drop of EdG was added to 2 mL of a solution of a preadjusted pH and KCl concentration inside the measurement cell. We verified that the results obtained were independent of the applied potential difference during the measurement (between 3 and 15 V cm^−1^), signaling that neither induced polarization nor non‐linear effects influenced the data. For the salt concentrations used in this work (larger than 1 mM), the Debye length *κ^−1^* is smaller than 10 nm. Thus, the product of particle size *a* by the inverse Debye length, *κa*, is greater than 100 and the Helmholtz‐Smoluchowski relation linking the electrophoretic mobility *µ* to the zeta potential is valid,^[^ ^16^ ^]^

(4) $$\mu \;=\frac{\varepsilon_{w}\cdot \varepsilon_{0}\cdot \zeta }{\eta }\;$$

where *ε_0_* is the electric permittivity of the vacuum and *ε_w_*, *η*, and are the relative permittivity and viscosity of the solution.

**Figure 4:**
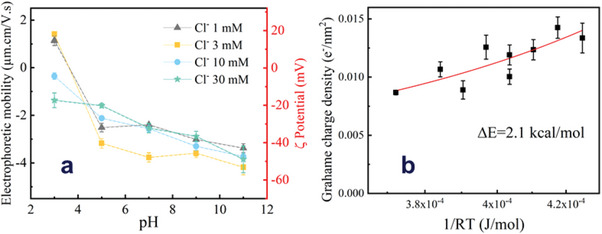
a) Electrophoretic mobility *µ* and *ζ* for graphene from EdG dispersed in KCl solutions at different pH and salt concentrations. b) Temperature dependence of graphene charge density calculated from measured *ζ*, using the Grahame equation as described in the text.

Measured electrophoretic mobility and *ζ* values calculated using Equation (4) are reported in **Figure** **4**. Each curve in this figure can be considered as a hydroxide‐ion adsorption isotherm. Several trends emerge from the measured data. First, *µ* is strongly dependent on the pH, decreasing when pH rises. Thus, water ions are potential‐determining ions, as observed for many types of interfaces. Second, for low ionic strength, a sign reversal of *µ* was observed at a particular pH value (the isoelectric point iep), ca. pH 4. Interestingly, at low salt concentrations, the reversal pH was close to the equivalence point observed by titration. The observed iep is a function of KCl concentration, shifting toward lower pH values for higher salt concentrations. This finding strongly suggests a certain degree of interaction of the graphene flakes with the chloride anions. Third, the curves measured for the different salt concentrations appear to intersect at pH ca. 4.5. Finally, little dependence on added salt is observed at basic pH. This is somehow surprising: it is commonly observed that, at constant pH, *ζ* decreases with increasing ionic strength due to increased electrostatic screening. This points again to the influence of the interaction of Cl^−^ ions with graphene, which counterbalances the effect of enhanced screening at high salt concentrations.

The typical *ζ* values observed at basic pH conditions are significantly more negative than the values derived from streaming potential measurements. This discrepancy could be due to the difference between the techniques (supported, one‐sided exposed graphene in streaming potential measurements, versus free‐floating graphene flakes in electrophoresis), or the effect of the oxygenated outer layer in the graphene deposit, as discussed above. However, it is important to highlight that there is a significant difference in the typical shear rates involved in each experiment. For the streaming potential tests, shear rates up to 10000 s^−1^ are commonly achieved for pressure differences of a few tens of mbar. On the contrary, typical shear rates during electrophoresis measurements rarely exceed 50 s^−1^, for the highest applied fields. Thus, if the apparent graphene surface charge corresponds to adsorbed ions (instead of structural charges) it is conceivable that the adsorbed ions (and not only the counterions) will be dragged by the water flow in the streaming potential tests, resulting in less negative values. We will come back to this important point later.

The variation of *ζ* pH just described suggests a significant interaction of graphene with water ions. To explore this interaction, we have measured the influence of temperature on the electrophoretic mobility of graphene, at fix OH^−^ concentration (0.16 mM, corresponding to pH 10.2 at 25 °C), in the absence of salt. Temperature was varied between 10 and 50 °C in a non‐sequential manner, to avoid systematic errors or long‐time spurious effects. To convert the measured mobility to *ζ*  values, the effect of temperature on the properties of the aqueous environment (viscosity and permittivity) was taken into account (Equation (4)). We found a small but consistent decrease *ζ*  with increasing temperature. We can estimate the surface charge density, *σ*, from the measured *ζ*, by using the Grahame equation,^[^ ^30^, ^31^ ^]^

(5) $$\sigma \;=\sqrt{8.c_{0}.\varepsilon_{w}.\varepsilon_{0}.k_{B}.T}\;\sinh\frac{e.\psi_{0}}{2.k_{B}.T}$$

where *c_0_* is the ionic bulk concentration, *e* is the electron charge, and ψ_0_ *t*he surface potential. This equation results from the necessary condition of global electroneutrality of the system. As has been extensively described in the literature, the electrokinetic *ζ*, measured at the (vaguely defined) slip plane, does not correspond to the actual surface potential ψ_0_. However, we can have an idea of the temperature dependence of the charge density, if we replace ψ_0_ by the experimentally accessible *ζ*  in Equation (5). As can be observed in Figure 4b, there is a slight decrease in charge density when the temperature is increased, suggesting a weak adsorption interaction. Assuming that the surface density of adsorbed charges is proportional to [OH^−^], σ = *K(T)*.[OH^−^] (Henry's law), the change of *K(T)* with reciprocal temperature allows a direct calculation of the adsorption enthalpy by using the van’t Hoff equation,

(6) $$\frac{d\ln\;K}{\mathrm{dT}}=\frac{\Delta H_{0}}{R.T^{2}}\;$$

which can be integrated as $K\left(T\right)\;=K_{0}\;e^{\Delta H_{0}/RT}$, where *ΔH_0_* is the standard enthalpy change of the adsorption. Following these steps, a value of 2.1 kcal moL^−1^ (ca. 3.5RT) can be estimated for the process of adsorption of OH^−^ on graphene, as indicated in Figure 4b, very close to the adsorption energy calculated by DFT (vide infra).

### 2.6 Mesoscopic Scale: EdG Raman Spectra

**Figure 5:**
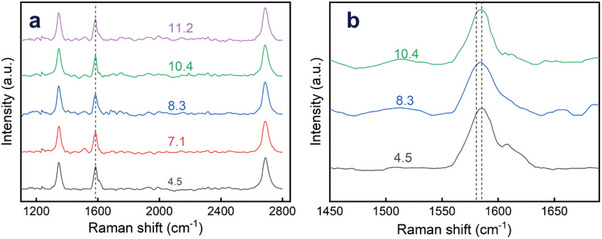
Raman spectra of EdG in water at different pH values. The vertical dashed line in a) shows the invariance of the G band with pH. (Raman spectra vs pH at 2400 lines/mm show the same invariance; supporting information Figure S7). Dashed lines in b) correspond to the change in the G band reported by Ushiba for pH between 4 and 9.^[^ ^23^ ^]^

Raman spectra of liquid EdG samples at different pH values were measured 24 h after adjusting the pH by adding small amounts of concentrated acid (HCl) or basic (KOH) solutions. The Raman spectrum of water itself has been shown to be independent of pH.^[^ ^32^ ^]^ Nondestructive Raman spectroscopy is a powerful technique for the characterization of changes in the electronic states and the energy band of graphene. It can be readily applied for the detailed characterization of EdG, as we have shown in the past.^[^ ^21^ ^]^ Typical Raman spectra are presented in **Figure** **5**. As can be observed in the Figure, narrow D, G, and 2D bands can be observed, evidencing the good quality of EdG. Interestingly, no significant changes are detected in the measured Raman spectra of EdG for the different pH investigated; the position of the different bands (and in particular the *G* band, ca. 1585 cm^−1^, and its right shoulder, the D’ band) appear to be independent of the pH of the EdG. This fact is quite significant, given that the position of the *G* band is a function of the degree of doping of graphene; shifts of several cm^−1^ in response to doping have been reported.^[^ ^21^, ^33^, ^34^ ^]^ The blue shift of the G band, as well as that of the 2D band has been attributed to slight n‐doping (4.10^12^ cm^−2^) and biaxial strain. In a study of graphene supported on Si/SiO_2_ substrates, Ushiba, and coworkers described a progressive upshift of the *G* band of ca. 5 cm^−1^ by changing the environmental pH from 9 to 4.^[^ ^35^ ^]^ As can be observed in Figure 5b, this does not seem to be the case for EdG: the position of the *G* band remains unchanged within the range of pH investigated. It is not clear if this apparent discrepancy is related to the use of buffer solutions in ref.[35] (we avoided the use of buffer solutions to keep the ionic environment as simple as possible), to the fact that the measured pH in EdG does not correspond to –log[H^+^] due to the interaction between water ions and the dispersed graphene, as discussed above, or to the material conditioning (supported vs dispersed graphene). Indeed, it has been shown that the substrate can have a significant influence on the state of charge of supported graphene.^[^ ^36^ ^]^ In any event, our results point to the fact that no variation in the degree of doping of graphene occurs when the pH of EdG is changed between 11 and 4.5, even though a substantial change in graphene electrophoretic mobility (and related zeta potential) was observed in the same pH range (cf. Figure 4). The pH‐induced change ζ does not appear to be accompanied by a significant variation in intrinsic graphene charge density. In agreement with this result, recent publications have reported little influence of pH on the response of graphene‐based transistors^[^ ^37^ ^]^ or on the charge neutrality point of graphene‐based electrodes^[^ ^38^ ^]^ for high‐quality (little defective) graphene. We will come back to this important point in the discussion.

### 2.7 Microscopic Scale: Deposit and Single Flake

**Figure 6:**
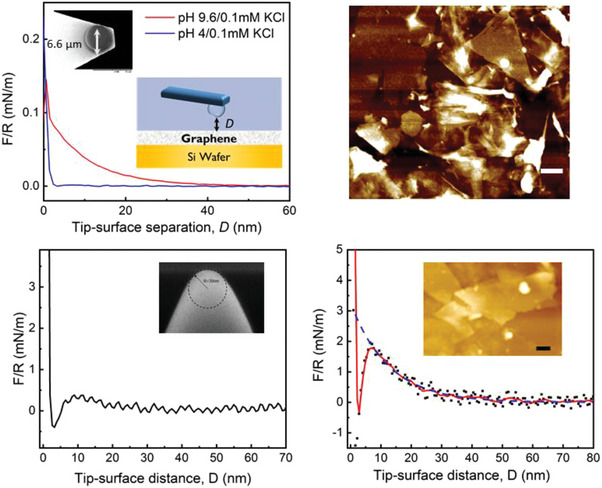
Atomic Force Microscope (AFM). Effect of aqueous pH on the probe‐graphene interaction force measured by AFM using a colloidal probe (upper‐left panel) and a 30 nm silica tip (lower‐left panel pH 3.8; lower‐right panel pH 9.8). Typical AFM height micrographs of the graphene deposit are shown in the right panel of the upper raw (scale bar 500 nm) and in the lower raw panel (scale bar 200 nm). The force profiles were obtained from the average of 100 independent tip‐substrate force‐distance curves (probe moving toward the sample). Equivalent results were obtained with 15 different graphene flakes in 3 different graphene deposits. MEB images of the 2 AFM probes are shown as insets.

As described in the experimental section, we measured the interaction forces between silica probes and graphene deposited on silicon wafers. Two different probe sizes were used: a relatively large silica colloidal probe (3.3 µm radii), and a smaller silicon AFM probe (30 nm radii, with a few nm thick native oxide layer). Larger and more accurate forces were obtained with the larger probe size, while local information on well‐defined graphene flakes was obtained from the latter. As the typical graphene flake size in our EdG samples is ca. 1 µm and the deposits were rather heterogeneous, the force measured with the larger probes explored an ill‐characterized geometry (typical AFM height micrographs of the deposits investigated are presented as insets in **Figure** **6**). On the contrary, when using the smaller probe, we could be sure that forces were measured in the middle of an individual graphene flake, sufficiently far from the edges to examine the interaction in the absence of the influence of the boundaries. As described in the experimental section, we have measured the forces at pH 9.6 (basic) and 4 (acid), ≈3 pH units apart from the neutral condition, to test the sign and reversibility of the charge of the graphene coating exposed to a relatively large amount of pH controlled water.

Typical results of the measured forces are presented in Figure 6. The results reported are the average of at least 100 individual force curves measured in different spots on the deposit or on a single flake. As could be anticipated, a much larger variability was observed for the smaller tip, which measures smaller forces (typical raw curves are presented in the supporting information, Figure S3, Supporting Information). Nevertheless, in both cases, there was a clear difference between the forces measured at acid and basic conditions. At basic pH, a repulsive, exponentially decaying force was observed at large tip‐substrate separations. On the contrary, very small interaction forces were observed at acidic pH down to separations of a few nm, when a strong repulsive interaction appears, due to the contact between the probe and the substrate. After normalization by the probe radius, which allows us to compare the results obtained between different probes, it is apparent that larger *F/R* values are measured with the smaller probe. This is understandable, given that the larger probe is interacting with a disordered, discontinuous deposit, and the flakes closer to the colloidal sphere will dominate the interaction force. For this reason, we did not attempt a quantitative interpretation of the *F/R* data measured with the colloidal probe. On the contrary, for the case of the smaller probe, a single flake is being probed, with an effective pristine area much larger than the tip size.

A crude description of the measured interaction can be developed in the framework of the Debye‐Huckel theory. At sufficiently large separations, the electrostatic interaction *F_elec_* dominates. For an asymmetric system, the variation of this force normalized by the radii of the probe *F/R* with the separation distance *D* can be calculated as,^[^ ^4^, ^31^ ^]^

(7) $$\frac{F_{\mathrm{elec}}}{R}=4\cdot \pi \cdot \varepsilon_{w}\cdot \;\varepsilon_{0}\cdot \kappa \cdot \psi_{1}\cdot \psi_{2}\cdot e^{-\kappa D}$$

where *κ* is the inverse of the Debye length, and *ψ*_i_ is the electrostatic potential of the 2 surfaces. As the surface potentials appear as a product in (7), it is not possible to independently determine their values from the measured forces alone. However, we can estimate the potential of graphene after making a reasonable guess of the electrostatic potential of the silica probe. It is well known that silica surfaces are negatively charged in water. Values between −50 and −70 mV, (obtained from interaction force experiments in symmetric, silica‐silica, conditions) have been reported.^[^ ^39^ ^]^ Thus, fitting the data measured with a single flake (small probe and well‐defined contact geometry) and using Equation (7), we can establish the limits for the electrostatic potential of graphene deposited from EdG (at basic pH conditions) between −65 and −85 mV. These values, corresponding to the potential close to the graphene‐liquid interface, are larger, but not incompatible with the *ζ* measured from electrophoresis, as *ζ* corresponds to the potential at an imprecisely defined “slip‐plane” a bit farther apart from the interface, as often described in the literature.^[^ ^16^ ^]^ In an extensive study with defect‐free graphene produced by chemical vapor deposition, Diao and coworkers reported similar values and pH‐dependence of the electrostatic potential of graphene in contact with aqueous solutions.^[^ ^40^ ^]^

All the measurements reported for the smaller probe were measured far from the graphene edges or from clear imperfections on the material, to minimize the potential influence of functional groups present in these regions. Nevertheless, no clear differences were detected between the forces measured in the middle of a flake and regions close to its edge. The general trends of the measured forces were robust and reversible. Quantitatively similar results were measured after repeated cycles of acidic and basic conditions, for the same surface‐probe pair. As pointed out above while discussing the streaming potential results, this reversibility discards the hypothesis that the negative charge of graphene in water is due to incomplete carbon oxidation upon transfer of THF solution to water. The negative charge of the graphene flakes appears to be an equilibrium condition and not a kinetically trapped state of unfinished oxidation. In addition, our results appear to rule out the influence of spurious functional groups on the observed graphene negative charge.

**Figure 7:**
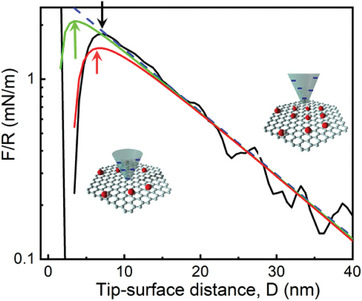
Normal Interaction force *F* between the AFM 30 nm silicon tip and graphene deposited from EdG, normalized by the tip radii *R* (black). Blue‐dashed line: exponential fit of the long‐range repulsive force. Green and red lines are calculated forces assuming additivity of electrostatic and dispersive forces, and values of the Hamaker constant of 2.73 × 10^−20^ and 1.5 × 10^−19 ^J respectively, as described in the text. The arrows signal the separations at which the mechanical instability (jump‐in) is observed (black) or would be expected (green, red). Insets illustrate the idea of charge regulation (OH^−^ desorption) upon the approach of the tip to the graphene plane.

Significantly, the measured force appears to follow a single‐exponential behavior, all the way down to small separations (ca. 7 nm), when a jump‐in into small separations (near tip‐substrate contact) is observed. We attempted to model the complete measured force profile using the Derjaguin−Landau−Verwey−Overbeeck theory (DLVO),^[^ ^16^, ^31^ ^]^ by combining the electrostatic repulsion (Equation 7) with a van der Waals (attractive) interaction term *F_vdW_*, which can be approximated as,

(8) $$F_{\mathrm{vdW}}=\;-\frac{A_{H}}{6.D^{2}}$$

where *A_H_* is an effective Hamaker constant for the silica/ graphene‐on‐silica interaction through water. As an example, the expected global interaction force, assuming *A_H_* = 2.73 × 10^−20^ J (as proposed by Diao and coworkers^[^ ^40^ ^]^), is presented in **Figure** **7** (green curve). As can be observed in the Figure, the expected jump‐in value should appear at significantly shorter distances than the ones observed by us (3.5 vs 7 nm). This discrepancy could be reduced by assuming (unrealistically) larger *A_H_* values (cf. red curve in the Figure, calculated assuming *A_H_* = 1.5 × 10^−19^ J). However, this would produce larger discrepancies with the measured data at medium‐range distances. A different interpretation of the measured interaction profile can be sketched in terms of a charge‐regulation scenario. As the negatively charged silica tip is approached to the graphene surface, the electrostatic energy cost increases. Thus, under these conditions, it may become preferable to adjust to a state of smaller charge density. This scenario appears plausible if OH^−^ adsorption is at the origin of the observed negative charge, but it is also compatible with a scenario of negative charge transfer from OH^−^ (or water) to graphene. This condition of charge regulation is also coherent with the idea of surface potential susceptible to the applied shear, probably at the origin of the quantitative difference between the *ζ* values measured by electrophoresis and streaming potential discussed before.

### 2.8 Nanometric Scale

**Figure 8:**
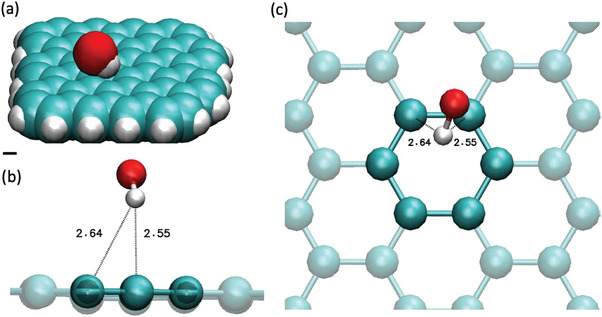
Snapshots of OH^−^ adsorption over a graphene flake obtained from DFT optimization. a) General view of the simulated system (atoms shown as spheres with Van der Waals radius). Scale bar 1 Å. Zoom (b) lateral view and c) top view) showing the adsorbed OH^−^ and the closest carbon atoms. The C atoms at distances less than 3 Å of the H atom of the OH^−^ are emphasized. The distances are indicated in Å. Color code C: cyan, O: red, H: white.

Our DFT calculations show that OH^−^ is attracted by a graphene flake in water, with an optimized geometry corresponding to an orientation with the H pointing toward 2 C (H‐C distance 2.6 Å) as shown in **Figure** **8** (see also additional details in the Supporting Information). The graphene flake is not substantially deformed. The obtained geometry does not depend on the different functionals and basis sets considered. The estimated adsorption energy (see Supporting Information) is −2.7 kcal moL^−1^. The estimated charge of the adsorbed OH^−^ is −0.98*e*, so we do not observe significant charge transfer from the ion to the graphene, in agreement with our conclusions from the Raman spectra. Interestingly, we observe excellent agreement between the interaction energy value experimentally determined from the temperature dependence of *ζ*, and the DFT calculations. In both cases, we can infer a weak, favorable interaction between the graphene plane and the hydroxide ions in water. On the contrary, the observed interaction between a hydrated proton and the same graphene flake is less significant: no dehydration of the Eigen cation is observed, even if the 2 species (graphene and cation) are close together (Figure S6, Supporting Information).

At this point, we can come back to the question of the origin of the charge at the graphene/water interface, which motivates this study. First, XPS and Raman data strongly suggest a minor presence of functional groups in the material. We have discussed these points in detail in the past.^[^ ^1^ ^]^ In addition, the sharp transition observed in the potentiometric titrations (cf. Figure 1) and the significant exothermic heat exchange observed by ITC (Figure 2) strongly argue against attributing the negative charge of graphene to the presence of ionogenic functional groups, which typically show more modest and broadly distributed enthalpy changes.^[^ ^28^ ^]^ Contrary to what is often observed in the case of rGO, in the case of good‐quality graphene in EdG the influence of functional groups can be ruled out. Second, the electrostatic and electrokinetic techniques used to characterize EdG (AFM, electrophoresis, and streaming potential), have all evidence that the charge of graphene is pH‐dependent. In addition, by cycling pH conditions we have shown that the degree of charge is reversible. Third, the absence of pH‐dependence of the *G* band in the Raman spectra and our DFT calculations show that the doping level of graphene in EdG is independent of pH, even as its electrophoretic mobility changes substantially. This result argues against the direct transfer of charge from water and water ions to the interface, an explanation advanced in recent studies of the charge of hydrophobic interfaces.^[^ ^17^, ^18^ ^]^ Thus, the only hypothesis that appears to be compatible with the set of data gathered in this study is the weak interaction of graphene with water ions, and in particular with OH^−^, as predicted by our DFT calculations. Favorable interaction between hydroxide ions and graphene (and exchange with protons at acidic pHs) would explain the observed pH‐dependent electrophoresis mobility and streaming potential, and the interaction forces measured by AFM. In addition, the reversible, weak OH^−^ adsorption is compatible with the charge‐regulation picture observed by AFM. The scenario of preferential interaction of graphene with hydroxide ions also explains the significant effect of graphene on pH measurements of EdG described before, as OH^−^ ions interacting with graphene will be rather undetectable by the glass membrane of the pH‐meter electrode.

A large number of studies, mainly based on electrokinetic or depletion measurements, have concluded that hydrophobic‐water interfaces are negatively charged at pH above 4. Most of these studies have hypothesized that this charge is due to OH^−^ preferential positioning at the interface.^[^ ^24^, ^41^, ^42^, ^43^ ^]^ However, most advanced spectroscopic studies and many theoretical calculations have failed to support this hypothesis.^[^ ^44^, ^45^ ^]^ As an alternative hypothesis, recent studies have suggested direct charge transfer from water to the interface, as a possible explanation for the negative charges observed at the interface. However, our results show that in the case of graphene, no substantial charge transferring is occurring. On the contrary, they point to the fact that the remarkable colloidal stability of graphene in water is due to its interaction with OH^−^ ions. This hypothesis has been evoked in the past to explain the charge and aqueous colloidal stability of nanoemulsions,^[^ ^24^ ^]^ and other carbon forms, like fullerenes,^[^ ^46^, ^47^ ^]^ carbon nanotubes,^[^ ^48^ ^]^ diamond,^[^ ^49^ ^]^ graphite,^[^ ^50^ ^]^ and is supported by ab initio molecular dynamics simulations.^[^ ^51^ ^]^ The ensemble of the results described in this work, and in particular the electrokinetic data and DFT calculations, suggests a weak attractive interaction between OH‐ ions and graphene, enabling electrostatic stabilization of graphene aqueous dispersions.

## 3 Conclusion

Graphene‐water interface is electrically charged. The broad evidence presented in this paper from a variety of different techniques shows that the favorable interaction between hydroxide ions and graphene is the reason for this charge, which allows the preparation of additives‐free, stable aqueous graphene dispersions. A posteriori, this fact is not enigmatic, and indeed, it becomes flawless in hindsight: charged interfaces are the rule, and non‐charged interfaces are clearly the exception.

## 4 Experimental Section

### EdG Preparation

The aqueous graphene dispersions (EdG) were prepared by liquid exfoliation of a graphite intercalated compound (GIC), KC_8_, as described before.^[^ ^1^, ^2^, ^3^ ^]^ This compound was synthesized by mixing molten potassium (Sigma‐Aldrich) and graphite flakes (Ausbury Carbons) at C/K = 8 (with 3% excess potassium) at 180 °C under an inert atmosphere for 5 h. The GIC was then exfoliated in distilled anhydrous THF at room temperature, under an inert atmosphere, and constant stirring at 230 rpm using a glass‐covered stirring bar for 5 days. Large undissolved particles of KC_8_ were then separated from the graphene solution by centrifugation. The obtained solution of graphenide in THF remains stable for many months if kept under an inert atmosphere, indicating that it was a thermodynamically stabled system. This graphenide solution, containing exfoliated and negatively charged graphenide flakes, was then exposed to oxygen, and the oxidized graphenide became neutral graphene dispersed in THF. This dispersion was then transferred to water and, after evaporation of the THF at atmospheric pressure and room temperature, a dispersion of neutral graphene in water (EdG) was obtained. Discussion of the extent of oxidation (electron removal) of graphenide to graphene^[^ ^6^, ^52^, ^53^ ^]^ and the related system of carbon nanotube to nanotubes^[^ ^54^ ^]^ has been amply discussed in the literature showing that any remaining electrons on the carbon nanoform were quenched by oxygen and or water.

### EdG Deposit Preparation

Graphene deposits were obtained by dip‐coating SiO_2_ substrates at the interface of an organic dispersion of graphene in THF and hexane, following a modified version of the method developed by Zarbin and coworkers.^[^ ^55^ ^]^ 20 mL of graphene dispersion in THF obtained by oxidation of a solution of graphenide in THF with dry air, was mixed with 40 mL of water. These 60 mL of dispersion were then mixed with 10 mL of hexane. The mixture was energetically stirred for a few seconds. After phase separation, a graphene film was formed at the interface between THF/water and hexane. The SiO_2_ substrate was placed beforehand horizontally below the interface. After the formation of the film, the substrate was slowly raised to recover it. After visually checking that most of the surface of the substrate was covered with graphene, the samples were air‐dried. Graphene deposits prepared by this method were between 40 and 100 nm thick (Figure S4, Supporting Information).

### EdG Characterization: XPS

The surface and in‐depth chemical analysis of EdG deposits were studied by X‐ray photoelectron spectroscopy (XPS) using a K‐Alpha spectrometer (ThermoFisher Scientific) equipped with a monochromatic Al‐*Kα* X‐ray source (1486.6 eV) with a beam diameter of 400 µm (surface points) or 200 µm (sputtered areas). Survey scans were collected in full energy range (0–1100 eV) at a pass energy of 200 eV, whereas high‐resolution spectra were obtained at a pass energy of 20 eV. The XPS depth profile was obtained through Ar^+^ ions sputtering using 500 eV low mode and 500 microns raster width. The estimated sputtering rate was 0.3 nm^−1^s on a 100 nm SiO_2_ thin layer.

### pH and Potentiometric Titration

The titration of 50 mL of EdG (ca. 0.5 g L^−1^ of graphene in water) was performed using a solution of 0.04 mol L^−1^ hydrochloric acid. The acid solution was gradually added to the EdG under constant stirring and the pH was monitored after each addition with a pHmeter (Consort C533 digital pH/mV meter). An interval of 30 to 60 s was imposed between successive additions, to ensure pH stabilization. Thermo Scientific Buffer Solutions (pH 4.01, 7.00, and 10.01) were used to calibrate the response of the glass membrane electrode. The same calibration procedure was implemented for all the pH data reported in this work. The titrations, as performed, were not reversible: significant destabilization of the graphene dispersion was observed for pH values below 3.

### Isothermal Titration Calorimetry, ITC

We have used ITC to quantify the heat exchange upon the pH change of EdG samples. Typically, a titration curve was obtained by adding aliquots of one system (the titrant) to a volume of a second system (the titrand) in an isothermal cell.^[^ ^28^, ^56^ ^]^ Simultaneously, a similar volume was injected on a second (reference) cell, but in the absence of one of the reacting species. ITC allows us to determine very precisely the heat exchange resulting from the interaction between titrant and titrand, by keeping the temperature constant in both cells and measuring (and compensating) the rate of heat production of each cell. For the isotherms reported in this work, 30 injections of 8 µL of titrant (EdG or basic water) to 950 µL of titrand (acid water), every 5 min were performed. This technique to explore the neutralization of EdG under different conditions was used.

### Streaming Potential

Aqueous solutions of different pH and ionic strengths were streamed through channels bounded by graphene‐coated silicon wafers. If the surfaces were not electrically neutral, the pressure‐driven flow induces convective charge transport of the counterions close to the walls, leading to the emergence of an electric field that opposes the current. The potential difference associated with this field was called streaming potential *ΔE_S_*. This resulting potential was measured at different pressure drop values (ZetaCAD, CAD instruments), and the zeta potential ζ was then calculated from the slope of the linear curves of streaming potential as a function of ΔP using the Smoluchowski equation^.[^ ^16^ ^]^ SiO_2_ substrates on which graphene was deposited as described above, using 1 mM KCl aqueous solutions adjusted to pH 9.6 and 4.1 by adding small aliquots of concentrated solutions of sodium hydroxide and hydrochloric acid were investigated. In this way, similar ionic strength was preserved in the different measurements. The effect of successive pH changes, to investigate the reversibility of the pH‐induced changes in zeta potential was explored.

### EdG Zeta Potential

EdG was diluted in aqueous solutions of different sodium chloride concentrations (0 to 30 mM) and adjusted to different pH (3 to 11) by adding the necessary amount of concentrated NaOH and HCl solutions. The electrophoretic mobility of the graphene flakes was measured (Zetasizer NanoZS, Malvern) and translated to zeta potential using the Smoluchowski equation.^[^ ^16^ ^]^

### Raman Spectroscopy

Raman measurements or EdG samples were performed with a HORIBA Xplora spectrometer, using a quartz cuvette containing the EdG samples, a holographic grating (1200 lines mm^−1^), and a 2.33 eV laser. Raman spectra of deionized water were also collected to obtain the water baseline, which was subtracted from the EdG spectra. The procedure for Raman data collection and treatment for EdG samples in the past has been extensively described.^[^ ^21^ ^]^

### Atomic Force Microscopy

Interaction forces between a graphene‐coated SiO_2_ substrate and a negatively charged AFM tip immersed in basic or acidic solutions were determined by atomic force microscopy AFM,  using 2 different probes: silica colloidal probes sQUBE CP‐PNPL‐SiO‐C (3.3 µm radii) on silicon nitride triangular cantilevers 200 µm long, and smaller silicon probes (Bruker, SAA‐HPI‐30; 30 nm end radii) on silicon nitride cantilevers. To study the interaction between the AFM tip and the graphene in solution, tip‐graphene approach‐separation cycles while monitoring the cantilever deflection were performed. The point of contact between the tip and the surface, when the deflection increased sharply and linearly with surface displacement, was identified. This linear response range was used to calibrate the response of the photodiode for each force curve, enabling us to determine the relative surface‐tip separations following well‐established methods.^[^ ^57^ ^]^ Here again, the graphene‐coated SiO_2_ substrate and the negatively charged AFM silica tips^[^ ^57^, ^58^, ^59^ ^]^ were first immersed in basic, then acidic, and then again in basic solutions. These solutions were 0.1 mM potassium chloride, adjusted to pH 9.6 and 4 using concentrated solutions of potassium hydroxide and hydrochloric acid.

### DFT Calculations

We have employed Gaussian 16^[^ ^60^ ^]^ to perform DFT calculations (geometry optimizations and energy calculations) of a graphene flake (≈1.3 nm) in water with and without adsorbed OH^−^. The graphene flake has 58 carbon atoms and it was capped with 20 hydrogen atoms in the edges. Water was modeled implicitly using the IEFPCM formalism.^[^ ^61^ ^]^ The DFT calculations were performed at 3 different levels of theory: B3LYP/6‐31G*, B3LYP/6‐311G*, and wB97XD/6‐31G* to assess the dependence of the results on the function employed. The double hybrid wB97XD functional^[^ ^62^ ^]^ employed was a dispersion‐corrected highly transferable functional that could predict accurate energetics for both bonded and non‐bonded interactions.

## Conflict of Interest

The authors declare no conflict of interest.

## Supporting information

Supporting Information

## Data Availability

The data that support the findings of this study are available from the corresponding author upon reasonable request.
