# Supplementary material for: Graphene in Water is Hardly Ever Neutral
Source: Adv Sci (Weinh). 2024 Aug 19;11(39):2403760. doi: 10.1002/advs.202403760 (PMC11497011; doi:10.1002/advs.202403760)
Supplement: Supplementary file 1 — Supporting Information [file ADVS-11-2403760-s001.pdf]

ADVANCED SCIENCE
Open Access

Supporting Information

for *Adv. Sci.*, DOI 10.1002/advs.202403760

Graphene in Water is Hardly Ever Neutral

Luna Boulbet-Friedelmeyer, Gilles Pécastaings, Christine Labrugère-Sarroste, Jordi Faraudo, Alain Pénicaud and Carlos Drummond**

Supporting information for: Graphene in water is hardly ever neutral

Luna Boulbet[1], Gilles Pécastaings[1], Christine Labrugère-Sarroste[2], Jordi Faraudo[3], Alain Pénicaud[1], Carlos Drummond[1]

[1]*Univ. Bordeaux, CNRS, CRPP, UMR 5031, 33600 Pessac, France* alain.penicaud@crpp.cnrs.fr, carlos.drummond@crpp.cnrs.fr

[2]*Carbon Waters, 14 avenue Pey Berland, 33600 Pessac, France*

[3]*Université de Bordeaux, CNRS, PLACAMAT, UAR 3626, F-33600 Pessac, France*

[4]*Institut de Ciència de Materials de Barcelona (ICMAB-CSIC), Campus de la UAB, E-08173 Bellaterra, Spain*

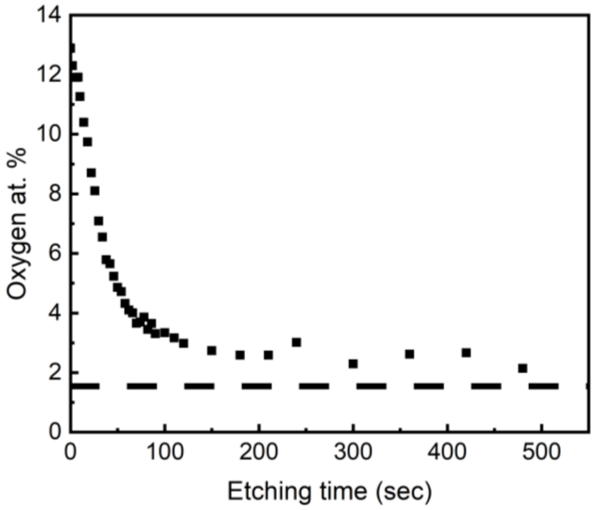

Figure S1. X-ray photoelectron spectroscopy. Oxygen content (at. %) as a function of etching time. After removing the outermost layer on the graphene deposit, the oxygen content is very close to the measured on the graphite starting material (dashed line), indicating little functionalization on the prepared graphene

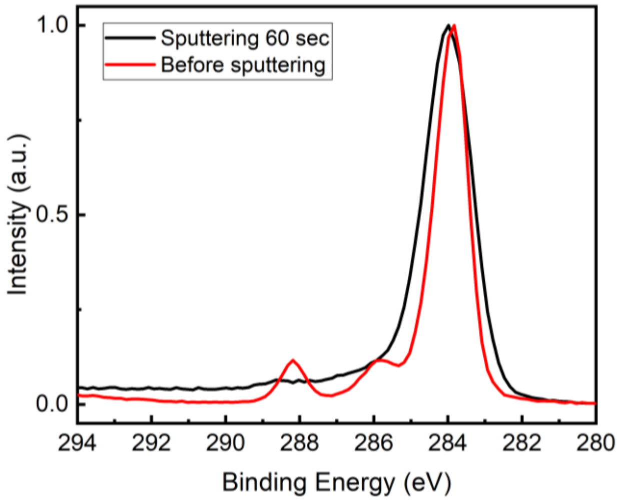

Figure S2. X-ray photoelectron spectroscopy. C 1s spectrum of EdG deposit. Red: as deposited. Black: 60 seconds Ar^+ ions sputtering. The high energy peaks in the 285-290 eV range on the red spectrum can be attributed to sp^3 carbon functionalization.[1]

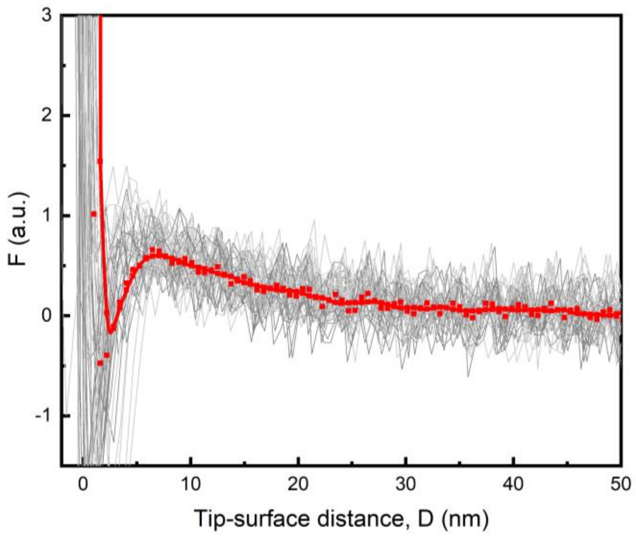

Figure S3: Example of measured AFM force profile. The red curve was obtained from the average of 100 independent tip-substrate force-distance curves (probe moving towards the sample). Equivalent results were obtained with 15 different graphene flakes in three different graphene deposits

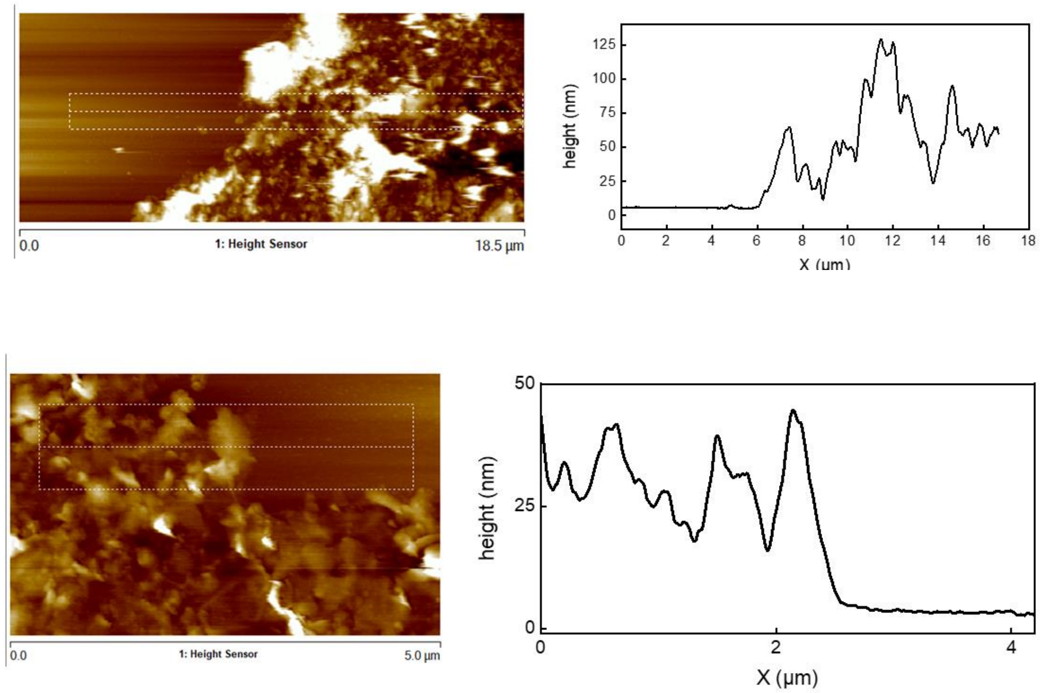

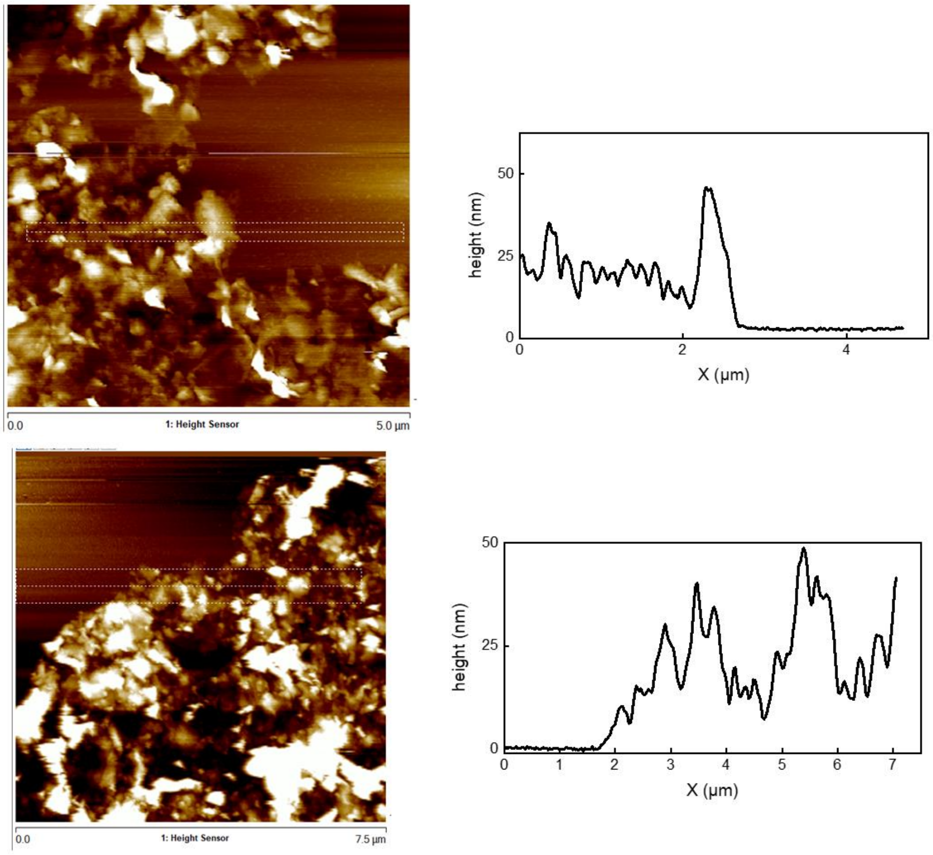

Figure S4: Typical AFM height micrographs and step profiles measured on samples of graphene coated on silicon wafers. The profiles, measured at the edges of the deposits, show that the graphene coatings are thicker than 30 nm.

Further details for DFT Calculations

Adsorption Energy calculations

As explained in the main paper, the DFT optimizations were performed at three different levels of theory: B3LYP/6-31G*, B3LYP/6-311G*, and wB97XD/6-31G*. In each case, we have considered the geometry optimization and energy calculation of three structures: a single graphene sheet (system 1), a single OH^- (system 2), and a graphene sheet with adsorbed OH^- (system 3). The adsorption energy was calculated as:

E_{ads} = E(system 3) – E (system 1) – E(system 2)

The obtained results were E_{ads} = -3.5 kcal/mol for B3LYP/6-31G*, E_{ads} = -3.9 kcal/mol for B3LYP/6-311G*, and E_{ads} = -2.7 kcal/mol for wB97XD/6-31G*. In the main paper, we reported the latter result for the adsorption energy (it corresponds to the highest level of theory between the three calculations). The optimized geometry obtained in the three levels of theory was almost indistinguishable. The geometry reported in the main paper corresponds to the wB97XD/6-31G* case.

Alternative configurations

In addition to the geometry obtained directly in the Gaussian 16 optimization as described in the main paper, we have considered an alternative possibility with the oxygen atom of the OH^-

oriented towards the graphene (instead of the hydrogen atom), as proposed in Ref.[2] This new configuration was initially generated by rotating the OH^- manually and it was later refined by an additional geometry optimization using the three levels of theory described above. In the three cases, we obtained a geometry as shown in Figure S1. The graphene sheet is deformed and the carbon atom closest to the OH^- is displaced about 0.4 Å in the perpendicular direction. In this configuration, the graphene flake is subject to a large stress which does not seem realistic. The configuration is also not consistent with the absence of dependence of the Raman spectra with pH described in the main paper.

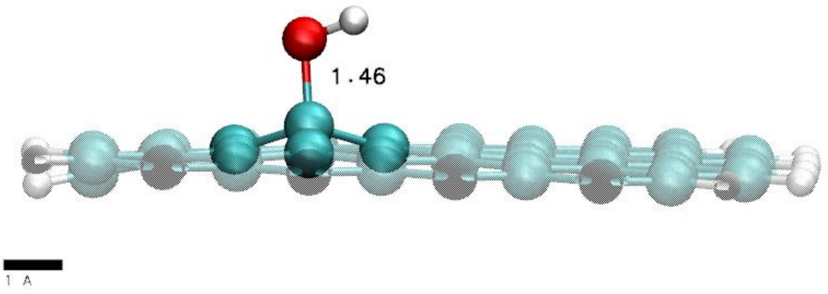

Figure S5: Image of the structure obtained by placing the OH- with the oxygen atom initially oriented towards the graphene flake, followed by DFT optimization at the wB97XD/6-31G* level of theory (scale bar 1 Å). The C atoms at distances less than 2.5 Å of the O atom of the OH^- are emphasized. The distances are indicated in Å. Color code C: cyan, O: red, H: white.

Hydrated proton close to the graphene surface

We have also considered the behavior of a hydrated proton near the graphene flake. In this case, we have considered a DFT optimization using wB97XD/6-31G* and the same implicit water model as before.

We have placed a H_3O^+ cation surrounded by 3 water molecules (the so-called Eigen ion $H_9O_4^+$) close to the same graphene flake described before in this section. The optimized configuration is shown in Figure S6; it is highly reminiscent of the results obtained previously for metal surfaces (see for example Fig. 10 in ref. [3]). Two of the water molecules of the cluster are adsorbed onto the surface and the hydrated proton links these molecules with two more water molecules not in contact with the surface.

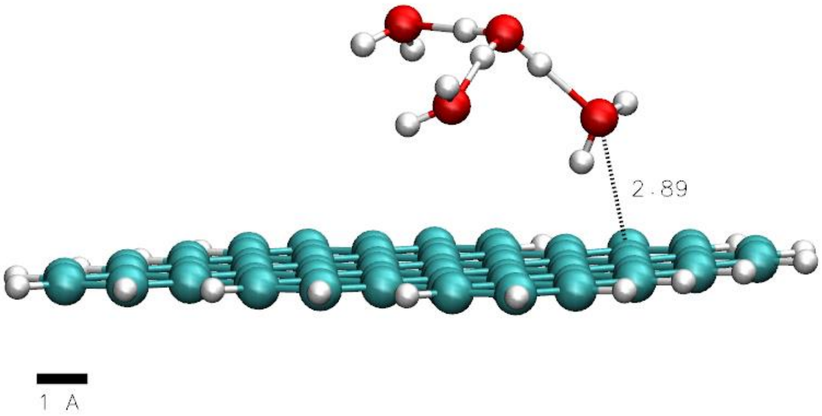

Figure S6: Image of optimized structure of an hydrated proton close to a graphene flake (scale bar 1 Å). The distances are indicated in Å. Color code C: cyan, O: red, H: white.

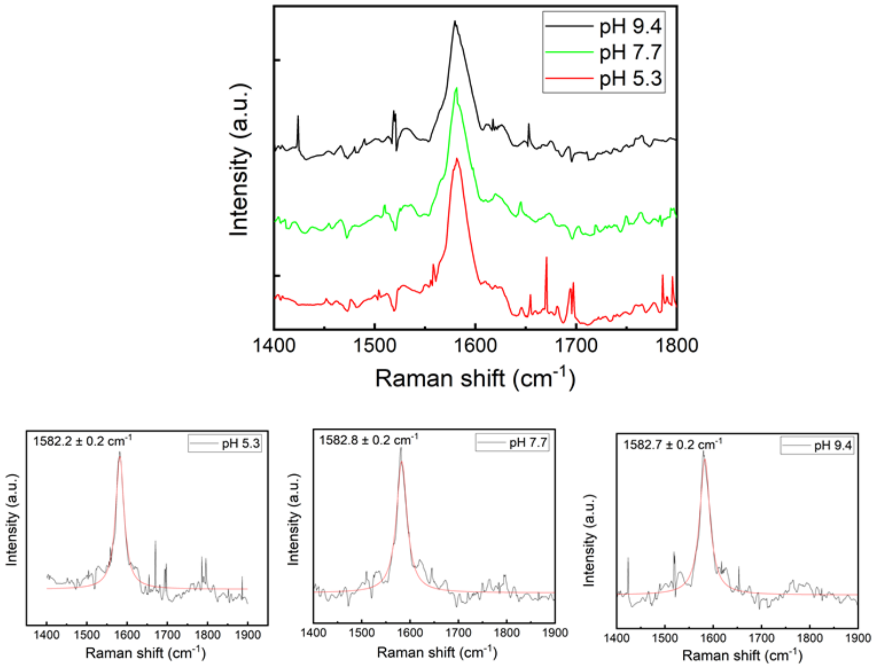

Figure S7: Raman spectra of EdG in water at different pH values. Measurements performed with a HORIBA Xplora spectrometer, using a quartz cuvette, a holographic grating (2400 lines/mm), and a 2.33eV laser. The Position of G band appears independent of changes of pH

REFERENCES

[1] H. A. Becerril, J. Mao, Z. Liu, R. M. Stoltenberg, Z. Bao, Y. Chen, *ACS Nano* **2008**, *2*, 463–470.

[2] B. Grosjean, C. Pean, A. Siria, L. Bocquet, R. Vuilleumier, M. L. Bocquet, *J. Phys. Chem. Lett.* **2016**, *7*, 4695–4700.

[3] P. Quaino, N. B. Luque, G. Soldano, R. Nazmutdinov, E. Santos, T. Roman, A. Lundin, A. Groß, W. Schmickler, *Electrochim. Acta* **2013**, *105*, 248–253.
